# Efficacy on the rocks: how hazardous drinking contextualizes the path from internalized heterosexism to antiretroviral adherence via adherence self-efficacy

**DOI:** 10.1093/abm/kaag019

**Published:** 2026-05-06

**Authors:** Stephen D Ramos, Wilson Vincent, Daniel E Siconolfi, Keith J Horvath, Chadwick K Campbell, Susan M Kegeles, Erik D Storholm

**Affiliations:** Department of Psychology, California State University, 5500 University Parkway, San Bernardino, California 92407, United States; Department of Psychology, Temple University, 1801 N Broad St, Philadelphia, Pennsylvania 19122, United States; RAND 1776 Main Street, Santa Monica, California 90401, United States; Department of Psychology, San Diego State University, 5500 Campanile Drive, San Diego, California 92182, United States; Herbert Wertheim School of Public Health & Human Longevity Science, University of California San Diego, 9500 Gilman Drive, La Jolla, California 92093, United States; School of Medicine, University of California San Francisco, 513 Parnassus Ave, San Francisco, California 94143, United States; School of Public Health, San Diego State University, 5500 Campanile Drive, San Diego, California 92182, United States

**Keywords:** alcohol, internalized heterosexism, adherence, self-efficacy, African American, HIV

## Abstract

**Background:**

Young Black sexual minority men living with HIV (YBSMM+) in the US South face a confluence of structural and psychosocial barriers that impact antiretroviral therapy (ART) adherence. Internalized heterosexism and hazardous drinking have emerged as significant yet understudied factors influencing adherence self-efficacy, a known determinant of sustained ART adherence.

**Purpose:**

This study examined the relationships among internalized heterosexism, adherence self-efficacy, and ART adherence in a sample of YBSMM+, with a particular focus on the moderating role of hazardous drinking.

**Methods:**

Utilizing cross-sectional survey data from a community-based sample of YBSMM+ in the US South, we conducted moderated mediation analyses to assess the direct and indirect effects of internalized heterosexism on ART adherence via adherence self-efficacy, as well as whether hazardous drinking moderates the direct effect of internalized heterosexism on adherence self-efficacy.

**Results:**

Findings indicated that higher levels of internalized heterosexism were associated with lower adherence self-efficacy, which in turn predicted lower ART adherence. Adherence self-efficacy significantly mediated the relationship between internalized heterosexism and ART adherence, replicating prior research. Moreover, participants who reported hazardous drinking evidenced a stronger inverse association between internalized heterosexism and adherence self-efficacy than those who did not report hazardous drinking.

**Conclusions:**

The results underscore the complex interplay among internalized heterosexism, adherence self-efficacy, and hazardous drinking in shaping ART adherence among YBSMM+. Interventions targeting adherence self-efficacy and addressing hazardous drinking, while also directly addressing internalized stigma, may help buffer the harmful effects of these factors and support improved HIV health outcomes in this population.

## Teaser

For young Black sexual minority men living with HIV in the US South, stigma related to sexual orientation was linked to lower confidence in taking HIV meds and more missed doses, particularly among those with heavy drinking. Addressing both stigma and alcohol use may help support better HIV health.

## Introduction

Despite a 12% decrease in new HIV infections since 2018, Black and African American individuals remain disproportionately affected, accounting for 37% of new cases while only representing approximately 14% of the US population.^1^^,^^2^ Among Black and African Americans, men aged 13-34 who have sex with men and those living in the US South experience significantly higher HIV rates than their racial/ethnic and geographic peers.^1^ Black and African American men living with HIV have lower rates of CD4 and viral load testing and reduced rates of viral suppression compared to the average for their respective age group.^3^ Moreover, only 51% are linked to care within 1 month of diagnosis, and just over half achieve viral suppression within 6 months.^3^ The low levels of HIV care engagement have long-term health consequences, increasing the risk of drug resistance that limits treatment effectiveness, accelerating disease progression and sequelae, and reducing future treatment options.^4^ Additionally, low engagement increases the likelihood of HIV transmission to partners due to the lack of viral suppression.^5^ Socio-structural factors, such as stress arising from discrimination and socioeconomic marginalization, are associated with mental health burdens and subsequent behavioral factors that increase HIV transmission risk (ie, substance use, condomless sex, reduced adherence to medication) among Black and African American sexual minority men.^6-8^ While structural interventions are urgently warranted, more data are required to better understand the pathways by which the socio-structural inequities influence HIV transmission risk and identify possible mechanisms for effective intervention.

Heterosexism is an experienced inequity where heterosexist beliefs, norms, and values are embedded and perpetuated by societal systems, institutions, and policies that create disadvantages for sexual minority individuals.^9^ Internalized heterosexism is a form of internalized stigma^10^ in which one adopts societal norms and negative attitudes toward one’s sexual minority orientation.^11^^,^^12^ Prior research has found Black youth to hold less positive attitudes toward sexual minority orientation than White youth on average^13^ and that these views are more likely to remain stable over time.^14^ Many Black youth who begin to explore or disclose their sexual identity report internalizing these negative attitudes, contributing to greater internalized forms of self-stigma and related mental health challenges.^15^ Specifically, higher levels of internalized heterosexism have been linked to behaviors that increase the risk for HIV transmission.^16-18^ For YBSMM, internalized heterosexist ideologies can shape perceptions about HIV and others among peers (eg, viewing HIV as a marker of moral failure or sexual deviance; anticipating judgment or rejection from other sexual minority peers),^19^ potentially exacerbating internalized stigma (eg, shame, self-blame, and anticipated HIV- or sexuality-related stigma)^10^ and leading to psychological distress,^20^ reduced self-acceptance,^21^ and greater reluctance to take HIV medications.^22^^,^^23^ Thus, existing literature suggests that HIV medications can function as symbolic reminders of negative societal schemas that frame HIV as a marker of moral failure, sexual deviance, or social devaluation, as well as internalized beliefs involving shame, self-blame, and anticipated stigma related to both HIV status and sexual orientation, thereby contributing to medication avoidance and non-adherence.^15^^,^^22^^,^^24^^,^^25^

Adherence self-efficacy, or the belief in one’s ability to follow prescribed medication regimens despite obstacles, is critically related to ART adherence.^26-30^ Specifically, higher levels of adherence self-efficacy are associated with better medication adherence and improved health outcomes among people living with HIV.^26-32^ Research has shown adherence self-efficacy to be an underlying mechanism that mediates the association between internalized stigma and ART adherence among individuals new to HIV care.^33^ This may be especially critical for marginalized populations, such as YBSMM+, who contend with additional stressors related to stigma, discrimination, and socioeconomic disadvantages^34^ that may undermine confidence in managing their treatment regimens.^32^ As such, understanding the context in which adherence self-efficacy effectively mediates the association between internalized forms of stigma (eg, internalized heterosexism) and ART adherence is essential for continued intervention development and targeted implementation of interventions to enhance self-efficacy.

Stress coping strategies can also greatly affect health. For example, alcohol use is often cited as a means of alleviating stress stemming from stigma and discrimination related to race, sexual minority status, and HIV among a variety of sexual minority men, including Black and African American sexual minority men.^35-38^ Research has also linked alcohol use to poorer overall engagement in HIV care among YBSMM+^39^ and Black sexual minority men living in the South, specifically.^40^ According to Cognitive Escape Theory, chronic stressors like discrimination and stigma based on sexual orientation can motivate individuals to use substances and alcohol to distance themselves (ie, escape-based coping) from distressing thoughts.^41^ This may relate to societal expectations of heteronormativity^42^ and hegemonic masculinity,^43^ which are associated with internalized stigmas.^44^^,^^45^ Hazardous drinking, a drinking quantity or pattern that places an individual at increased risk of adverse health events, is often used in an attempt to cope with these distressing internal states and represents a significant clinical threshold of disordered use, distress, and impairment.^46^^,^^47^ This complex interaction between sociocultural contexts, distressing internal states, and health behaviors is particularly evident in YBSMM+, where alcohol use is linked to lower adherence to ART.^38^^,^^40^^,^^48^^,^^49^ This may be partially explained by alcohol’s influence on cognitive and belief-based processes central to the HBM. Here, hazardous drinking can increase perceived barriers to ART by shaping expectations about harm or reduced benefit when medication is taken in the context of alcohol use (eg, alcohol-ART toxicity beliefs),^50-52^ while also diminishing adherence self-efficacy through impaired self-regulation.

Related to internal cognitive processes, research shows that internalized homonegativity can reduce confidence in managing HIV treatment, which undermines medication adherence and increases viral load, particularly among individuals who also engage in heavy drinking.^53^ This suggests that the relationships between hazardous drinking and adherence self-efficacy are complex and may be linked via adherence to ART medications, especially within the contexts of internalized stigmas like internalized heterosexism. However, given that this research was conducted in Uganda, experiences likely do not generalize to YBSMM+ living in the US South and requires further investigation. The Health Belief Model (HBM) is a widely used framework for understanding health behaviors, emphasizing how individuals’ perceptions of susceptibility, severity, benefits, barriers, self-efficacy, and cues to action shape decision-making around care.^54^ Viewed through the HBM, internalized heterosexism and alcohol use as a coping strategy may shape beliefs about HIV care by increasing perceived barriers to ART and undermining adherence self-efficacy, while simultaneously reducing perceived susceptibility to viral rebound through stigma-related beliefs about inevitability, deservingness, or diminished concern for the consequences of nonadherence. For example, internalized heterosexism may foster stigma-related beliefs that frame HIV-related health consequences as inevitable or less personally consequential, leading individuals to perceive viral rebound as expected rather than preventable and thereby reducing perceived susceptibility to the consequences of nonadherence or hazardous alcohol use. Within this model, they can also lower the perceived benefits of strict dosing, raise everyday barriers to taking pills, and weaken the cues that prompt on-time use. These shifts can persist beyond drinking episodes, fueling nonadherence and stabilizing maladaptive beliefs that undermine consistent ART use.

Together, the frameworks are complementary: Cognitive Escape Theory can contextualize why YBSMM+ may drink to distance themselves from stigma-related distress, while the Health Belief Model clarifies how drinking, and even the anticipation of drinking, may shape adherence-relevant beliefs and behaviors not attributable to intoxication alone. Given the negative downstream impact on biological markers of HIV health (ie, viral load, CD4 count) attributed to poorer ART adherence, this study aims to contextualize the relationship between internalized heterosexism, adherence self-efficacy, and ART adherence among a sample of YBSMM+. To do so, this study investigates how hazardous drinking may influence the strength of adherence self-efficacy as an underlying mechanism linking internalized heterosexism to ART adherence. By contextualizing these mechanisms, this research seeks to inform targeted interventions that enhance adherence self-efficacy and mitigate the adverse impact of internalized heterosexism and alcohol use on HIV treatment outcomes within this population.

## Methods

### Participants and procedures

Data for this study were drawn from the fourth wave of data collection (Wave 4) from the Texas Young Black Men’s Health Survey, a longitudinal community-based cohort of YBSMM+ recruited from 2 cities in Texas (Dallas and Houston) that focused on factors relating to HIV care engagement. Wave 4 data collection occurred between May 2022 and October 2022 from a study site affiliated with the University of Texas Southwestern in Dallas or from a local community-based organization affiliated with the University of Texas, Houston. For baseline enrollment in this study, participants were required to be 18- to 29-year-old men residing in the greater Dallas or Houston metropolitan areas who identified as Black or African American, were assigned male gender at birth, reported sex with a man in the past year, and were willing to get tested for HIV. Those whose HIV test was reactive at baseline were enrolled in the longitudinal cohort study.

As part of this larger parent study, participants completed approximately yearly surveys surrounding factors relating to HIV care engagement. Full methodological details surrounding respondent-driven sampling, study compensation, and confirmation of HIV status are described elsewhere.^39^ Informed consent was obtained from all participants in this study.

## Measures

### Sociodemographic characteristics

Participants were asked a series of questions regarding their background characteristics, including age, education, employment, income, insurance coverage, and prior history of incarceration.

### Internalized heterosexism

The Internalized Heterosexism Scale^55^ is adapted from Nungesser’s examination of “antihomosexual” attitudes^56^ and measures current internalization of societal norms and negative attitudes toward one’s own sexual orientation through five items. Three items are reverse-coded, and the overall measure of internal consistency indicates Cronbach α = .80 for this sample. Items are scored on a 5-point Likert scale, ranging from “Not at all” to “A great deal,” with higher composite scores indicating higher levels of internalized heterosexism. Sample items include, “Does having sex with other men make you dislike yourself?”

### Adherence self-efficacy

The HIV Treatment Adherence Self-Efficacy Scale (HIV-ASES) is a 12-item scale assessing one’s confidence to carry out important treatment-related behaviors related to adhering to treatment plans, including medication regimens across the past month.^57^ Sample items include “How confident are you that you can stick to your treatment plan when side effects interfere with daily activities?” Responses were collected on a 5-point Likert scale ranging from “Not at all confident” to “Completely confident,” with higher composite scores indicating higher adherence self-efficacy. Analysis of internal consistency showed Cronbach α = .97 for this sample.

### Antiretroviral therapy adherence

In keeping with prior Black SMM cohort work that uses a single brief adherence outcome item and SMM surveillance methods (eg, 30-day 100% dose adherence),^48^^,^^58^ we assessed adherence with a 1-item self-report of days per week participants missed at least 1 dose of ART in the prior 60 days. We measured ART adherence only among men who were actively engaged in ART treatment, thereby excluding those not currently on ART. This approach conceptually distinguishes our analysis of adherence behaviors from broader engagement in ART care. Responses to this item were collected using a 6-point Likert scale ranging from “Never” to “Every day.” Lower scores indicate more favorable adherence. Although subgroup-specific criterion validation against viral load among Black SMM is limited, extensive validation in broader cohorts of people living with HIV supports the accuracy of single-item self-report measures for identifying clinically meaningful nonadherence.^59-62^

### Hazardous drinking

Hazardous drinking use was measured using the Alcohol Use Disorders Identification Test-Concise (AUDIT-C), a brief alcohol screening instrument that reliably identifies hazardous drinking within the prior 60 days.^63^ The AUDIT-C is a 3-item scale that uses a 5-point Likert rating, scored 0-12. Scores ≥4 indicated a positive screening for hazardous drinking (1 = yes; 0 = no), which may also be an indication of an alcohol use disorder. AUDIT-C scores are only available at the fourth wave of data collection. Analysis of internal consistency showed Cronbach α = .74 for this sample.

## Analytic plan

Specific variables of interest were available only at the final wave of data collection as part of the larger parent study. As such, all data used are from Wave 4 of the data collection period. We calculated descriptive statistics using measures of central tendency (mean and standard deviation) and frequency with percentages to characterize our sample (*N* = 118) using IBM SPSS v. 28.0.1.^64^ Adopting listwise deletion for complete-case analyses, a total of *n* = 15 participants were excluded from the inferential analyses, yielding *N* = 103 as the analytic sample.

Within our analytic approach, it is important to note that the ordering of constructs reflects their relative stability, conceptual distance from behavior, and the phenomenological sequence through which these processes are experienced, rather than assumptions about temporal change or data collection timing. Internalized heterosexism is widely conceptualized as a relatively stable, though modifiable, psychosocial construct shaped across the life course through repeated socialization experiences and minority stress exposure; phenomenologically, it constitutes a background orientation through which individuals interpret identity, self-worth, and social contexts.^65^ As such, it is expected to exhibit greater temporal stability than more proximal cognitive or behavioral constructs and is appropriately specified as the antecedent in the model. In contrast, adherence self-efficacy reflects individuals’ contemporaneous evaluations of their capacity to adhere to treatment and conceptually precedes ART adherence by shaping motivation, confidence, and perceived behavioral control.^57^ ART adherence is the most behaviorally proximal construct and is operationalized as a current outcome reflecting sustained behavior over a clinically meaningful time window, as longer adherence windows more accurately capture adherence as a lived behavioral pattern and are more predictive of virologically relevant outcomes than brief or momentary assessment periods.^66^ Thus, the specified mediation pathway reflects the lived psychological ordering of meaning, belief, and action, grounded in construct stability and phenomenological precedence among concurrently assessed variables, rather than temporal ordering inferred from measurement timing.

**Figure 1:**
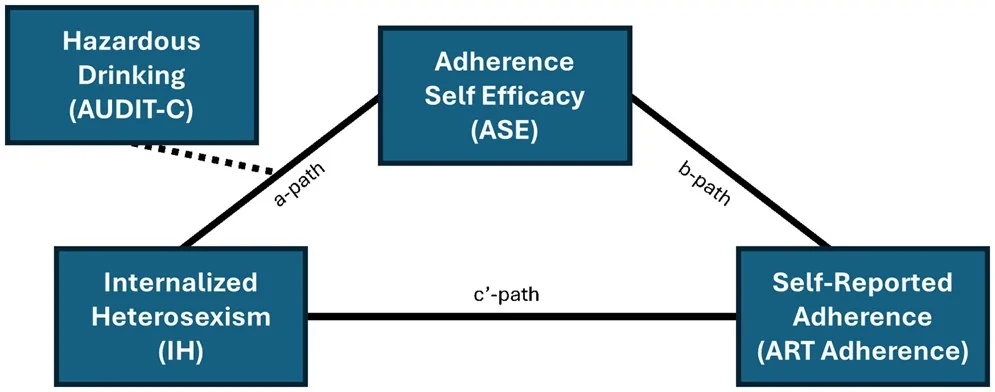
Hypothesized moderated mediation model for testing.

For the primary analyses, we conducted a moderated mediation analysis using the Hayes PROCESS Macro for SPSS.^67^ For this analysis, we used Model 7 (Figure 1), using a percentile bootstrap estimation approach with 10 000 samples, using AUDIT-C scores as a dichotomous moderator (positive screen vs. negative screen) within this model to allow for evaluation of adherence self-efficacy as a mediator between internalized heterosexism and self-reported adherence, moderated via the presence or absence of hazardous drinking. This approach collapses individuals who do not use alcohol into the same category as individuals with non-hazardous levels of use. To support this approach, we ran a sensitivity analysis, using 3 separate alcohol drinking groups (no consumption, non-hazardous, hazardous) as a group-level moderator of the mediation analysis. Results paralleled the results of the simpler dichotomous moderator model. See Table S1. For the final model, we adopted the simpler dichotomous moderated mediation model and present confidence intervals with decimals to the ten-thousandth place to prevent rounding-based misinterpretations. To interpret the presence of moderated mediation, we examine the index of moderated mediation, as recommended by Hayes,^67^ while emphasizing that these findings should be considered tentative and exploratory due to the cross-sectional nature of the data and lack of temporal order of variables, despite support in health behavior theory.

## Results

### Descriptive statistics

A total of 331 men were enrolled in the larger parent study. Of these, *n *= 166 discontinued their participation in the time between enrollment (ie, Wave 1) and the current (ie, Wave 4) data collection. An additional *n *= 46 participants were excluded from the current analyses because they were not currently receiving ART treatment, and 1 additional participant was excluded for declining to respond to the ART adherence measure. The final derived sample (*N *= 118) had a mean age of 30.56 years (SD = 2.80). Nearly half of the participants had some college or an associate’s or technical degree education (48.8%), with smaller proportions having a high school or GED education (35.5%) and a bachelor’s degree (7.6%). More than half were employed full-time for wages (56.1%), and nearly 90% of participants reported an income of <$60 000. Most had insurance coverage (73.1%) and had not been incarcerated in the prior year (91.0%), while approximately a third reported never missing an ART dose (33.1%) or missing a dose <1 time a week (29.7%). Approximately 30% screened positive for hazardous drinking. For full descriptive statistics, see Table 1.

**Table 1:** Descriptive statistics.

Sociodemographic	*n*	%	*M*	SD	Min	Max
**Age (years)**	118	—	30.39	2.76	20	36
**Internalized heterosexism**	110	—	10.71	4.89	6	24
**Treatment adherence self-efficacy**	112	—	47.06	8.80	13	55
**Highest education completed**						
** Did not complete high school**	5	4.2	—	—	—	—
** High school diploma/GED**	44	37.3	—	—	—	—
** Some college/AA/technical**	55	46.6	—	—	—	—
** Bachelor’s degree**	10	8.5	—	—	—	—
** Any graduate studies**	4	3.4	—	—	—	—
**Employment status**						
** Employed full-time**	71	60.2	—	—	—	—
** Employed part-time**	15	12.7	—	—	—	—
** Unemployed**	29	24.6	—	—	—	—
** On disability**	3	2.5	—	—	—	—
**Annual personal income**						
** Less than $10 000**	20	16.9	—	—	—	—
** $10 000-$19 999**	18	15.3	—	—	—	—
** $20 000-$39 999**	32	27.1	—	—	—	—
** $40 000-$59 999**	25	21.2	—	—	—	—
** $60 000-$79 999**	10	8.5	—	—	—	—
** $80 000-$99 999**	1	0.8	—	—	—	—
** $100 000 or more**	1	0.8	—	—	—	—
** Don’t know**	9	7.6	—	—	—	—
** Decline to answer**	2	1.7	—	—	—	—
**Health insurance coverage**						
** Yes**	89	75.4	—	—	—	—
** No**	27	22.9	—	—	—	—
** Decline to answer**	2	1.7	—	—	—	—
**Past year: jail/prison/juvenile detention**						
** Yes**	1	0.8	—	—	—	—
** No**	117	99.2	—	—	—	—
**Missed ART dose frequency (past 60 days)**						
** Every day**	12	10.2	—	—	—	—
** 4-6 days per week**	3	2.5	—	—	—	—
** 2-3 days per week**	15	12.7	—	—	—	—
** Once per week**	14	11.9	—	—	—	—
** <Once per week**	35	29.7	—	—	—	—
** Never**	39	33.1	—	—	—	—
**Hazardous drinking**						
** Negative screen (AUDIT-C score = 0-3)**	77	68.1	—	—	—	—
** Positive screen (AUDIT-C score >4)**	36	31.9	—	—	—	—

All variables are from Wave 4 of the parent study. ART adherence variables reflect adherence among participants actively engaged in ART treatment. Missing data were handled via listwise deletion, resulting in an analytic sample of *N *= 103 for inferential analyses.

### Moderated mediation analysis

We use a top-down approach to present the moderated mediation analysis. First, we examine whether the overall moderated mediation is significant. Then, we analyze the indirect effects. After that, we present the models for the individual paths: the a-path (IH → ASE), the b-path (ASE → ART adherence), and the c’-path (IH → ART adherence, accounting for the indirect effect through ASE). Here, the index of moderated mediation was significant (*b *= *−*0.04, 95% CI: −0.0900 to −0.0015), indicating that the strength of the mediation is significantly moderated by the severity of alcohol use. Specifically, the mediated effect of internalized heterosexism on ART adherence through adherence self-efficacy was found to be stronger among participants who reported hazardous drinking (*b = *−0.07, 95% CI: −0.1217 to −0.0213) compared to when alcohol use was absent or non-hazardous (*b = *−0.03, 95% CI: −0.0546 to −0.0073). Significant interactions were observed in the a-path (*b = *−0.67, *P* < .05, Δ*R*^2^ = 0.03), with internalized heterosexism more strongly reducing adherence self-efficacy among men reporting hazardous drinking (*b = *−1.14, *P* < .001) than in those who did not report hazardous drinking (*b = *−0.47, *P* < .01). The b-path (*b* = 0.06, *P* <.01) was also significant; however, the direct effect (c’-path) was not significant (*b = *−0.03, *P* >.05). For full regression results see Table 2 below.

**Table 2:** Regression results for model paths.

Variable	Model a-path			Model b/c’-path		
	*b*	SE	*P*	*b*	SE	*P*
**IH**	−.47	.18	<.001	−.03	.03	>.05
**AUDIT-C**	1.20	3.82	>.05			
**IH × AUDIT-C**	−.67	.32	<.05			
**ASE**				.06	.02	<.01

*N* = 103. Model for a-path *R*^2^ = .30, *F*(3, 99) = 14.33, *P* < .001. Model for b-path and c’-path *R*^2^ = .13, *F*(2, 100) = 7.56, *P* <.001. The model a-path column indicates associations to ASE as the regression outcome. The model b/c’-path column indicates associations to ART Adherence as the regression outcome.

## Discussion

The findings of this study underscore the complex interplay among internalized heterosexism, adherence self-efficacy, and hazardous drinking in shaping ART adherence among YBSMM+ in the US South. Consistent with prior research, our results indicate that internalized heterosexism is associated with lower adherence self-efficacy, which is a key mechanism for sustained ART adherence.^26-30^ Additionally, our study replicates findings that adherence self-efficacy mediates the association between internalized stigmas and ART adherence.^33^ Importantly, we build upon this literature by demonstrating that hazardous drinking, which is a well-established risk factor for adverse physical health outcomes, significantly amplifies the detrimental effects of internalized heterosexism, a key psychosocial factor, on both adherence self-efficacy and ART adherence.

YBSMM+ often navigate intersecting sources of stress arising from discrimination, socioeconomic marginalization, and broader systemic-level injustices.^38^^,^^68^^,^^69^ Among Black SMM, higher levels of internalized stigma are linked to greater hazardous drinking, consistent with alcohol use as a coping response to stigma-related stress,^70^ aligning with the theoretical foundations of Cognitive Escape Theory.^41^ Hazardous alcohol use, in turn, is linked to poorer ART adherence, partly via reductions in adherence self-efficacy,^53^ a central determinant of adherence among YBSMM+.^71^ Together, findings from this study and the existing literature suggest that hazardous drinking may likely function as a maladaptive strategy for managing internalized heterosexism, eroding adherence self-efficacy, and undermining ART adherence among YBSMM+.

However, the impact of alcohol may extend beyond the direct effects that alcohol shows on cognitive impacts and disrupted medication routines.^72-74^ Alcohol use may contribute to distorted adherence-related beliefs, including the misconception that consuming ART while drinking is harmful (ie, alcohol-ART interactive toxicity beliefs).^50^^,^^51^ Nested within the Health Belief Model,^54^ YBSMM+ who experience alcohol-ART interactive toxicity beliefs and do not adhere to their ART may perceive low susceptibility to viral rebound or have a lowered self-efficacy to adhere to their ART while consuming alcohol. For example, a study of people living with HIV in the southeastern U.S. found that beliefs about alcohol–ART toxicity were associated with increased intentional nonadherence, mediated by lower confidence (ie, self-efficacy) to take ART during drinking episodes, even after controlling for frequency of alcohol use and other barriers.^52^ As such, these alcohol-related cognitive and affective states may undermine adherence self-efficacy, sustaining a feedback loop in which uncertain confidence and maladaptive expectancies may perpetuate hazardous drinking and missed ART doses. These beliefs may lead to intentional nonadherence, not only on days when alcohol is consumed but also on days when the individual is abstinent from alcohol due to lingering concerns about potential harm.^50^^,^^51^ Given that social networks often play a central role in shaping both adherence beliefs and experiences of internalized heterosexism,^51^ it is likely that social reinforcement of alcohol-related misinformation and internalization of stigma further contribute to the observed patterns of nonadherence.^11^

Despite many socio-structural drivers of HIV-related inequities among this community, adherence self-efficacy is well known to associate with more optimal ART adherence,^75^ and is a common mechanism targeted by effective adherence interventions.^75^ Our findings suggest it may be a particularly meaningful target for YBSMM+ experiencing internalized heterosexism and hazardous drinking. Because adherence and alcohol use are socially reinforced,^76^^,^^77^ community- and peer-based interventions may be especially impactful. Interventions that develop skills to deconstruct hegemonic masculinity and internalized heterosexism, such as adaptations of the Mpowerment program^55^ or Critical Consciousness interventions,^78^ could be integrated into culturally grounded, community-adaptable alcohol reduction programs. Examples include adapted drink-refusal skills training,^79^ culturally congruent motivational interviewing interventions,^80^ and smaller group-based programs targeting substance use and sexual risk reduction.^81^ These empirically supported approaches not only reduce alcohol and substance use but can also be delivered preventively to decrease maladaptive coping while enhancing adherence self-efficacy through mastery experiences, peer modeling, and empowerment.

Group workshops that engage YBSMM+ in critical discussions on masculinity, sexuality, mental health, and social relationships may further build empowerment, reduce stigma, and strengthen skills that generalize to adherence-related tasks, such as communicating with providers, problem-solving dosing barriers, and planning for adherence during drinking episodes.^82^ By addressing socio-structural barriers and promoting peer support and navigation, such approaches may enhance treatment self-efficacy, a pathway shown to mediate improvements in HIV care outcomes.^83^ Future research should examine the efficacy of these interventions in improving adherence self-efficacy and, subsequently, ART adherence among YBSMM+ with hazardous alcohol use.

However, this raises the question of whether such interventions would be effective without explicitly addressing drinking behaviors. While the National Institute on Alcohol Abuse and Alcoholism (NIAAA) recommends Screening, Brief Intervention, and Referral to Treatment (SBIRT) as a meaningful strategy for addressing alcohol use,^84^ YBSMM+ are often marginalized within these traditional care settings. As such, SBIRT may not adequately reach this population. There is a pressing need first to understand the drinking environments of YBSMM+ who engage in hazardous drinking to inform community-based and community-integrated adaptations of evidence-based interventions. These adapted interventions should address hazardous drinking alongside the abovementioned psychosocial factors that influence ART adherence. Such an approach aligns with core principles of the person-centered recovery models within community mental health,^85^ which emphasizes a dual strategy: supporting individual recovery journeys while also fostering environmental conditions that enable and sustain recovery.

This study is limited by its cross-sectional design, which prevents causal interpretations of the relationships between internalized heterosexism, hazardous drinking, adherence self-efficacy, and ART adherence. In particular, the cross-sectional nature constrains the interpretation of our mediation analysis, as temporal ordering of the variables cannot be established. Thus, the identified pathways should be viewed as tentative and hypothesis-generating rather than confirmatory. The study is also subject to attrition over time, with approximately 50% of the baseline sample retained at Wave 4, which served as the analytic sample. The 5- to 6-year interval between Wave 1 and Wave 4 coincided with substantial external disruptions, including the COVID-19 pandemic, the Black Lives Matter movement, the killings of George Floyd and Breonna Taylor, and broader sociopolitical unrest, which likely contributed to challenges in participant retention. In addition, the sample comprised individuals living with a chronic illness, a population historically difficult to retain in longitudinal health and social science research due to health-related instability and competing life demands.

It is possible that participants retained at Wave 4 were, on average, healthier or experienced fewer social and structural barriers than those lost to follow-up. However, demographic and economic indicators presented in Table 1 demonstrate that retained participants nonetheless experienced substantial levels of economic hardship and social vulnerability. Thus, while attrition may limit generalizability, the findings provide meaningful insight into the experiences of individuals navigating ART adherence under conditions of significant adversity and should be interpreted with appropriate caution. Future studies would benefit from assessing these processes using shorter follow-up intervals and repeated measurement of key psychosocial and structural variables to reduce attrition and more fully capture change over time.

Finally, our reliance on self-reported measures may be susceptible to bias in reporting, particularly in assessing alcohol use and adherence behaviors. Future research should employ longitudinal designs and objective adherence assessments to better understand the temporal dynamics and causal mechanisms underlying these associations. Additionally, qualitative studies may provide deeper insight into the lived experiences of YBSMM+ navigating the intersection of internalized heterosexism, alcohol use, and ART adherence, helping to refine intervention approaches through more tailored and empathic approaches for the community.

In conclusion, our findings suggest that hazardous drinking may function as a coping response to internalized heterosexism, with potential downstream effects on adherence self-efficacy and ART adherence. These results highlight the value of further exploring multifaceted interventions that address both the psychological and behavioral drivers of nonadherence, in addition to hazardous drinking, as possible strategies to improve ART adherence and overall health outcomes for YBSMM+. While broader socio-structural antecedents of health inequity (eg, systemic heterosexism) are challenging to address directly, interventions targeting individual-level factors such as alcohol use and internalized heterosexism may represent promising avenues for future research and practice.

## Supplementary Material

kaag019_Supplementary_Data
