# Supplementary material for: Efficacy on the rocks: how hazardous drinking contextualizes the path from internalized heterosexism to antiretroviral adherence via adherence self-efficacy
Source: Ann Behav Med. 2026 May 6;60(1):kaag019. doi: 10.1093/abm/kaag019 (PMC13148247; doi:10.1093/abm/kaag019)
Supplement: kaag019_Supplementary_Data [file kaag019_supplementary_data.docx]

| Variable | Model a-path | | | Model b/c’-path | | |
| --- | --- | --- | --- | --- | --- | --- |
|  | *b* | *SE* | *p* | *b* | *SE* | *p* |
| IH | -.32 | .29 | >.05 | -.03 | .03 | >.05 |
| AUDIT-C (1) | 1.51 | 4.38 | >.05 |  |  |  |
| AUDIT-C (2) | 1.90 | 4.81 | >.05 |  |  |  |
| IH x AUDIT-C (1) | -.29 | .37 | >.05 |  |  |  |
| IH x AUDIT-C (2) | -.83 | .39 | <.05 |  |  |  |
| ASE |  |  |  | .06 | .02 | <.01 |

This approach treats AUDIT-C as a 3-category variable with “no consumption” as the reference category. AUDIT-C (1) indicates dummy coding of AUDIT-C values for consumption of alcohol that did not screen positive for hazardous drinking. AUDIT-C (2) indicates dummy coding of AUDIT-C values for scores indicating hazardous drinking.
